# Oesophageal artefact may significantly affect oesophageal pressure measurement in mechanically ventilated patients

**DOI:** 10.1186/cc14322

**Published:** 2015-03-16

**Authors:** F Mojoli, F Torriglia, M Pozzi, S Bianzina, G Tavazzi, A Orlando, A Braschi

**Affiliations:** 1 Fondazione IRCCS Policlinico S. Matteo Hospital - University of Pavia, Italy

## Introduction

Oesophageal pressure is increasingly used to monitor and manage mechanically ventilated patients. Even if the oesophageal balloon catheter is correctly positioned, the measurement can be affected by inappropriate balloon filling and/or oesophageal reaction to balloon inflation. We aimed to assess the oesophageal reaction to oesophageal balloon filling in mechanically ventilated patients.

## Methods

An oesophageal balloon catheter (NutriVent; Sidam, Mirandola, Italy) was introduced in mid/distal thoracic position in 31 patients under invasive mechanical ventilation for acute respiratory failure. At ambient pressure, the balloon of the NutriVent catheter can be inflated up to 6 ml without generation of recoil pressure. The balloon was progressively inflated in 0.5 ml steps up to 9 ml and end-expiratory values of balloon pressure were used to assemble the balloon pressure-volume curve. The minimum slope section of the curve was graphically detected and inflation volumes corresponding to this part of the curve were considered appropriate. Overdistension of the balloon being excluded by definition in this section of the curve, its slope was attributed to the oesophageal reaction to balloon inflation.

## Results

Forty-five oesophageal balloon pressure-volume curves were obtained in 31 patients undergoing controlled mechanical ventilation (PEEP 12 ± 5 cmH_2_O, FiO_2_ 0.7 ± 0.2, tidal volume/ideal body weight 8.0 ± 1.6 ml/kg). According to the graphically detected minimum slope section of the curve, the minimum and maximum appropriate balloon volumes were 1.5 ± 0.6 ml and 5.3 ± 0.9 ml, respectively. Between these two volumes, the slope of the curve was 1.1 ± 0.5 cmH_2_O/ml, ranging from 0.3 to 3.1 cmH_2_O/ml.

## Conclusion

The oesophageal artefact - that is, the reaction of the oesophageal wall to balloon inflation - may be clinically significant, being on average 1 cmH_2_O for each millilitre of volume injected in the catheter, but reaching values as high as 3 cmH_2_O/ml. The pressure generated by the oesophageal reaction leads to overestimation of pleural pressure. Therefore, the oesophageal artefact may significantly affect clinical decision-making based on absolute values of oesophageal pressure.

